# Current Evidence of the Oncological Benefit-Risk Profile of Hormone Replacement Therapy

**DOI:** 10.3390/medicina55090573

**Published:** 2019-09-07

**Authors:** Marta D’Alonzo, Valentina Elisabetta Bounous, Michela Villa, Nicoletta Biglia

**Affiliations:** Academic Division of Gynaecology and Obstetrics, Mauriziano Hospital, University of Turin, 10128 Turin, Italy

**Keywords:** menopause, hormone, women’s health, breast cancer, gynecological cancers, colon cancer

## Abstract

Hormone replacement therapy (HRT) remains the most effective treatment for menopausal symptoms and has been shown to prevent bone loss and fracture. The progestogen is added to provide endometrial protection in women with an intact uterus. After the publication of the initial WHI (Women’s Health Initiative) results in 2002 reporting an overall increased risk of breast cancer, many women discontinued HRT. Despite the re-analysis of the results by subgroups of patients and updates with extended follow-up, much controversy remains, which we will analyze later in the text. Different types of estrogen or progestogen, as well as different formulations, doses, and durations, may play a role in HRT’s effects on breast tissue. Evidence states that conjugated equine estrogen (CEE), compared to estro-progestin therapy, shows a better profile risk (HR 0.79, CI 0.65–0.97) and that, among different type of progestins, those structurally related to testosterone show a higher risk (RR 3.35, CI 1.07–10.4). Chronic unopposed endometrial exposure to estrogen increases the risk of endometrial hyperplasia and cancer, whereas the association with progestins, especially in continuous combined regimen, seems to reduce the risk (RR 0.71, CI 0.56–0.90). HRT was also associated with a protective effect on colon cancer risk (HR 0.61, CI 0.42–0.87). Data about ovarian and cervical cancer are still controversial.

## 1. Introduction

The estrogen deprivation following the menopause status may impact on several aspects of health and quality of life determining vasomotor symptoms (VMS), genitourinary syndrome of menopause (GSM), cognitive dysfunction, sleep disturbance, and changes in bone metabolism. 

Hormone replacement therapy (HRT) remains the most effective treatment for VMS and GSM and has been shown to prevent bone loss and fracture. A progestogen is added to provide endometrial protection in women with an intact uterus.

Despite this high prevalence of symptoms, it has been claimed that only 10–15% of women seeking medical help because of fear of the treatment and diffidence of clinicians in prescribing therapy [1].

After the publication of the initial WHI (Women’s Health Initiative) results in 2002 reporting an overall increased risk of breast cancer, heart disease, stroke, and venous thromboembolism, many women discontinued HRT. HRT prescriptions in the United States rapidly decreased over one year by approximately 40% down to 20% [2,3]. In a European survey published in 2016, 61% of women claimed they would not consider taking HRT because they were afraid of the increased risk of breast cancer (25%), cardiovascular disease (34%), and weight gain (26%) [4]. In other previously published surveys, the main reason for not using HRT was the fear of cancer (38%) [5].

The WHI remains the largest randomized controlled trial of HRT, but it only compared conjugated equine estrogens (CEEs) and medroxyprogesterone acetate (MPA) versus placebo in patients with an elevated average age (44% between 60 and 69 years old). The North American Menopause Society, on the contrary, suggests starting HRT before than 60 years or within 10 years of menopause onset because the benefit-risk ratio is most favorable. The design of the WHI led to an overestimation of the risks for women aged less than 60 years and at low risk of cardiovascular disease and breast cancer. Despite the reanalysis of the results by age and years since menopause and the updates with extended follow up, much controversy remains, which we will analyze later in the text. The cancer risk of HRT differs depending on many factors, so treatment should be individualized to identify the most appropriate dose, regimen, duration, and route of administration, using the best available evidence, with periodic reevaluation of the woman’s benefit-risk profile [1].

## 2. HRT and Cancer Risk

In a study published in 2017 by Simin et al., an increased risk of any cancer of 9% appears among hormone replacement therapy ever-users (SIR 1.09, CI 1.07–1.11). The risk results lower among estrogen-only use, and the risk increases with age, reaching a peak in women ≥70 years old. Stratifying by tumor type, the risk varies among female reproductive organ cancers (breast, endometrial, and ovarian); on the contrary, the risk of all gastrointestinal tract cancer is decreased by 10% in HRT users (SIR 0.9, CI 0.86–0.94). In the analysis by subgroups study, evidence shows that the risk changes depending on the type of formulation and regimen of therapy [6].

## 3. HRT and Breast Cancer 

Breast cancer is the most frequently diagnosed cancer and is the leading cause of cancer-related death in women. It affects up to one of eight women who survive up to the age of 85 years in Western countries. The disease reaches a peak of incidence in the 50–59 years age range [7]. 

The WHI study results suggested a breast cancer increase in HRT users (HR 1.26, CI 1.00–1.59), but no data about mortality were reported because of a short follow-up period (mean of 7.9 years). This risk, in absolute terms, corresponds to 9 additional breast cancers per 10,000 women using estrogen-progestin therapy for five or more years [8]. Breast cancer increase in HRT users had already been observed 20 years before, in 1997, in the Collaborative Group Study; this review of 50 observational studies evidenced a relative risk of 1.35 (CI 1.21–1.49) for women who had used HRT for five years or longer. No significant excess of breast cancer had been seen five or more years after cessation of HRT use or in relation to the duration of use [9].

The North American Menopause Society (NAMS) position statement of 2017 asserts that different types of estrogen or progestogen, as well as different formulations, doses, durations, times of initiation, and patient characteristics, may play a role in HRT’s effects on breast tissue. Other risk factors need to be also considered in prescribing HRT, such as BMI, cardiovascular diseases, and lifestyle factors including smoke, physical activity, and alcohol intake.

Anderson et al. randomized women aged between 50 and 79 years with prior hysterectomy to conjugated equine estrogen (CEE) therapy versus placebo. Patients receiving estrogen alone had a significant reduction in overall breast cancer incidence after an extended follow up of 11.8 years (HR 0.77, CI 0.62–0.95, *p* = 0.02), especially in infiltrating ductal carcinoma (HR 0.67, CI 0.51–0.88). No differences emerged for receptor-positive and -negative tumors. Results also showed a reduction in deaths for breast cancer in the CEE group (HR 0.37, CI 0.13–0.91, *p* = 0.03). However, the risk reduction was restricted to patients without benign breast disease (*p* = 0.01) and a family history of breast cancer (*p* = 0.02) [10].

The update of WHI trial outcomes published in 2013 with extended post-intervention follow-up of 13 years analyzed 27,347 patients treated with conjugated equine estrogen (CEE, 0.625 mg/day) with medroxyprogesterone acetate (MPA, 2.5 mg/day) or CEE alone. During the intervention phase, HR for breast cancer was 1.24 (CI 1.01–1.53) with CEE-MPA use and 0.79 (CI 0.62–1.02) with CEE use. Estrogen-progestin use significantly increased the risk of breast cancer during cumulative follow-up (HR 1.28, CI 1.11–1.48), even if a year-to-year reduction after stopping was observed. By contrast, the use of unopposed estrogen seemed to significantly reduce risk during cumulative follow-up time (HR 0.79, CI 0.65–0.97) in patients with an intact uterus [11]. Among estro-progestin regimens, cyclic therapy seems to have a better risk profile compared to continuous therapy [12,13,14].

Not all treatments are equal, and in the literature, some authors suggest a lower impact of transdermal estrogens rather than oral ones because of a difference in levels of estrone in breast tissue and serum. However, prospective randomized trials comparing the effects of oral and transdermal estrogens on breast cancer risk are lacking. In a sub-analysis of the Million Women Study, no statistically significant differences were found in relation to the route of administration (RR 1.63, CI 1.04–2.56, and RR 1.34, CI 1.08–1.66, for oral and transdermal, respectively) [15]. 

In combined estro-progestin therapies, the estrogen component is usually estradiol, but preparations may contain a large variety of progestins that may impact on breast cancer risk. Large epidemiological studies, such as the French E3N Study [12] and Cecile study [13], indicate that natural progesterone and dydrogesterone may be associated with a more favorable risk profile compared to the other progestins (RR 1.16, CI 0.94–1.43 versus RR 1.69, CI 1.50–1.91) [12]. In particular, those structurally related to testosterone show a higher risk (RR 3.35, CI 1.07–10.4) [13].

To avoid the proliferative effect of estrogen on breast tissue and the endometrium, a new class of molecules has been developed. TSECs (tissue-selective estrogen complexes) are a combination of estrogens and SERMs (selective estrogen receptor modulators): CE 0.45 mg plus Bazedoxifene 20 mg has been available in Italy since 2015. Bazedoxifene guarantees a safety profile on endometrium and operates as an ER antagonist and, in the breast, antagonizes estrogen-stimulated cell proliferation. This combination demonstrated effects on bone mineral density and VMS with a significant improvement in vaginal dryness, dyspareunia, and sexual function compared to placebo. In addition, Bazedoxifene does not affect age-related changes in breast density, suggesting a possible better profile on breast cancer risk compared to estro-progestin therapy, even if data from large randomized trials are lacking [16,17].

Breast cancer risk is also influenced by the duration of treatment and by the interval between menopause and the beginning of HRT. The increased risk is generally observed for five years use or longer and significantly decreases five or more years after cessation of HRT use [9]. Beral et al. demonstrated that risk of breast cancer is greater in current users (RR 1.68, CI 164–1.72) than in past users (RR 1.08, CI 1.04–1.12), and it declines rapidly after use ceased [15]. Several studies have reported that the time interval between the onset of menopause and the beginning of HRT treatment may influence breast cancer risk, with a higher risk in women who start HRT within one year after the onset of menopause [13,18].

Mammography density changes with age and in response to exposure to hormones. Increased breast density may influence mammographic interpretation and is more strongly associated with the risk of aggressive breast tumor subtypes, especially among postmenopausal women using combined estrogen plus progesterone therapy. The mechanism for the association between breast cancer risk and breast density is unknown, but recently, it was postulated to result from an increase in tissue synthesis of estrogen from aromatase in dense tissue [19,20]. In the WHI trial, the abnormal mammography rate that required further testing was higher among women treated with estrogens plus progestins than placebo: They had a 4% greater risk of having further investigation, such as biopsy, after five years (*p* < 0.001). Estrogens alone have a lower impact on mammography density. 

In support of the association between HRT and breast cancer risk, in a first analysis, the decline in breast cancer incidence starting in 2003 was attributed to HRT prescription reduction [21]. Wachtel et al. [22] analyzed the SEER (Surveillance, Epidemiology, and End Results) data to clarify this possible relationship. HRT prescription rates had a dramatic drop since 2002; instead, rates for both lobular and ductal breast cancer showed a decline that began before 2001 and then a subsequent increase after 2003, such that by 2012, rates were similar to those seen in 2001. This fluctuating trend indicates that there is not a clear relationship between HRT and breast cancer incidence [22,23,24]. 

## 4. HRT and Endometrial Cancer

Chronic unopposed endometrial exposure to estrogen increases the risk of endometrial hyperplasia and cancer. Progestin association is important in ensuring endometrial protection in women with an intact uterus. The previously cited study of Watchel et al. that analyzed the relationship between the HRT prescription and cancer incidence, highlights an increase in the incidence rate of endometrial carcinoma after the dramatic drop in HRT prescription consequent to WHI result publication: the 2012 rates were 1.46 times higher than those in 2001, whereas other study found only a change in trend for mortality but not in the incidence [22].

In the WHI trial, a small non-significant reduction in endometrial cancer risk was observed with estrogen plus progestin use in a continuous combined regimen (HR = 0.81; 95% CI 0.48–1.36) compared to a cyclic regimen. The reduction became significant in the post-intervention phase lasting up to 2010 (HR 0.58, CI 0.40–0.86) and after cumulative follow-up of 13 years (HR 0.67, CI 0.49–0.91) [11]. The Million Women Study confirmed these data in women using continuous combined preparations (RR 0.71, CI 0.56–0.90), whereas tibolone and estrogens alone were associated with an increased risk. Beneficial effects of combined HRT were greatest in obese women, who usually have a higher incidence of endometrial cancer than non-obese women [25]. In a recent review of 28 studies analyzing HRT in patients with an intact uterus, the use of estrogens alone, tibolone, and sequential combined therapy increased the risk of endometrial cancer, even when treatment lasted less than five years; continuous combined therapy seems to be risk-free [26].

## 5. HRT and Ovarian Cancer

The influence of estrogen in promoting the development of epithelial ovarian cancer is still controversial. The WHI study did not find an association of CEE+MPA use with an increased risk of ovarian cancer after a mean of 5.6 years (HR 1.41, CI 0.75–2.66) and neither after a 13 years cumulative follow-up (HR 1.24, CI 0.83–1.87) [11]. The study by Simin et al. confirmed these data with a non-significantly association in all HRT-users (1.09, CI 1.00–1.19) [6]. Otherwise, some studies have suggested a possible increased risk. In the Million Women Study, an increase in incidence for current users emerged with longer duration; the risk does not differ significantly by type of HRT or administration route. The risk varies significantly with histological type, being greater for serous than for mucinous, endometrioid, or clear cell tumors in current users [27]. A recent meta-analysis of 52 epidemiological studies revealed a significant increase of ovarian cancer risk in current HRT-users, even in those with less than five years use. In ex-users, risk decreased the longer ago HRT use had ceased, however, a small excess risk still seemed to be present 10 years after stopping HRT. Also in this study, risk varies substantially by tumor type, being increased only for the two most common histological types, serous (RR 1.53, CI 140–1.66) and endometrioid tumors (RR 1.42, CI 1.20–1.67) [28]. However, it should be considered that the prospective data in this meta-analysis are heavily influenced by the two major studies, the Million Women Study and the Danish Sex Hormones Register Study. The risk in absolute terms comes down to one excess case of ovarian cancer per 1000 users after five years and a very low value for women using HRT, and it may be beyond the capacity of a meta-analysis of observational studies to accurately predict the correct risk.

A study published in 2017 evaluated the association between HRT and serous borderline tumors: Compared with non-users, women using HRT had a statistically significant 32% increased risk of developing serous borderline tumors without a correlation with the duration of use [29].

## 6. HRT and Cervical Cancer

In literature, very few studies evaluate HRT and cervical cancer risk. In a large study on 308,036 women recruited in the EPIC (European Prospective Investigation into Cancer and Nutrition) cohort, a reduction in cervical cancer risk among peri- and post-menopausal women using HRT was found (0.5, CI 0.4–0.8), and the effect became stronger with longer duration of use (≥5 years 0.4, CI 0.2–0.9). The risk reduction of cervical cancer could be overestimated: One possible bias is that women taking HRT are more frequently screened than non-users, and they are being diagnosed and treated earlier. The intrinsic mechanisms that might explain the biology of these potential associations are currently unknown. Data from HPV transgenic mouse models suggest that estrogens promote cervical carcinogenesis and progesterone inhibits cervical cancer [30].

## 7. HRT and Colon-Rectal Cancer (CRC)

Although the WHI trial identified greater health risks than benefits among women in the HRT-users group, the use of estrogen plus progestin was associated with a significant decrease in the incidence of colorectal cancer. Women in the hormone group had fewer cases of cancer of all histologic types (HR 0.61, CI 0.42–0.87). In comparison between women taking CEE alone and placebo, no difference was found in the incidence of colorectal invasive cancer, thus the protective effect may be attributed to progestins. In the decade following the publication of the WHI trial, other studies confirmed these data. Predominantly, increasing benefits for CRC risk reduction was seen in current and recent HRT users. Questions remain as to how long the CRC prevention benefits of HRT persist after discontinuation of these therapies. In post-intervention and 13 years cumulative follow up of WHI study, the HRs were neutral [1].

A study by Simin et al. published in 2017 assessed that HRT ever-use is associated with a 10% decreased risk of all gastrointestinal cancers; in particular in liver and colon cancers, the reduction of risk is statistically significant (0.81, CI 0.65–0.99, for liver and 0.90, CI 0.84–0.95, for colon) [6]. A recent study in 2018 showed a reduction of risk of colorectal cancer (0.81, *p* = 0.005) and also a reduction in death from colorectal cancer (HR 0.63, *p* = 0.002) and all-cause mortality (0.76, *p* = 0.001) in HRT users [31]. A possible explanation of the influence of HRT on colon cancer is the predominance of receptors of estrogens in the colon, which seems to lose expression during carcinogenesis. In clinical models, estradiol and progesterone reduce proliferation and increase apoptosis in colon tumors, probably through estrogen receptor activation [32].

Although there are protective effects, the balance of risks and benefits does not make HRT suitable for primary prevention in the general population.

## Abbreviations

HR	Hazard Ratio
RR	Relative Risk
SIR	Standardized Incidence Ratio
CI	Confidence Interval
